# Supplementary material for: Signal peptide represses GluK1 surface and synaptic trafficking through binding to amino-terminal domain
Source: Nat Commun. 2018 Nov 19;9:4879. doi: 10.1038/s41467-018-07403-7 (PMC6242971; doi:10.1038/s41467-018-07403-7)
Supplement: Supplementary file 1 — Supplementary Information [file 41467_2018_7403_MOESM1_ESM.pdf]

Signal peptide represses GluK1 surface and synaptic trafficking through binding to amino-terminal domain

Duan et al.

Supplementary Information

Supplementary Figures 1-4

Supplementary Table 1   Expression Constructs Primers

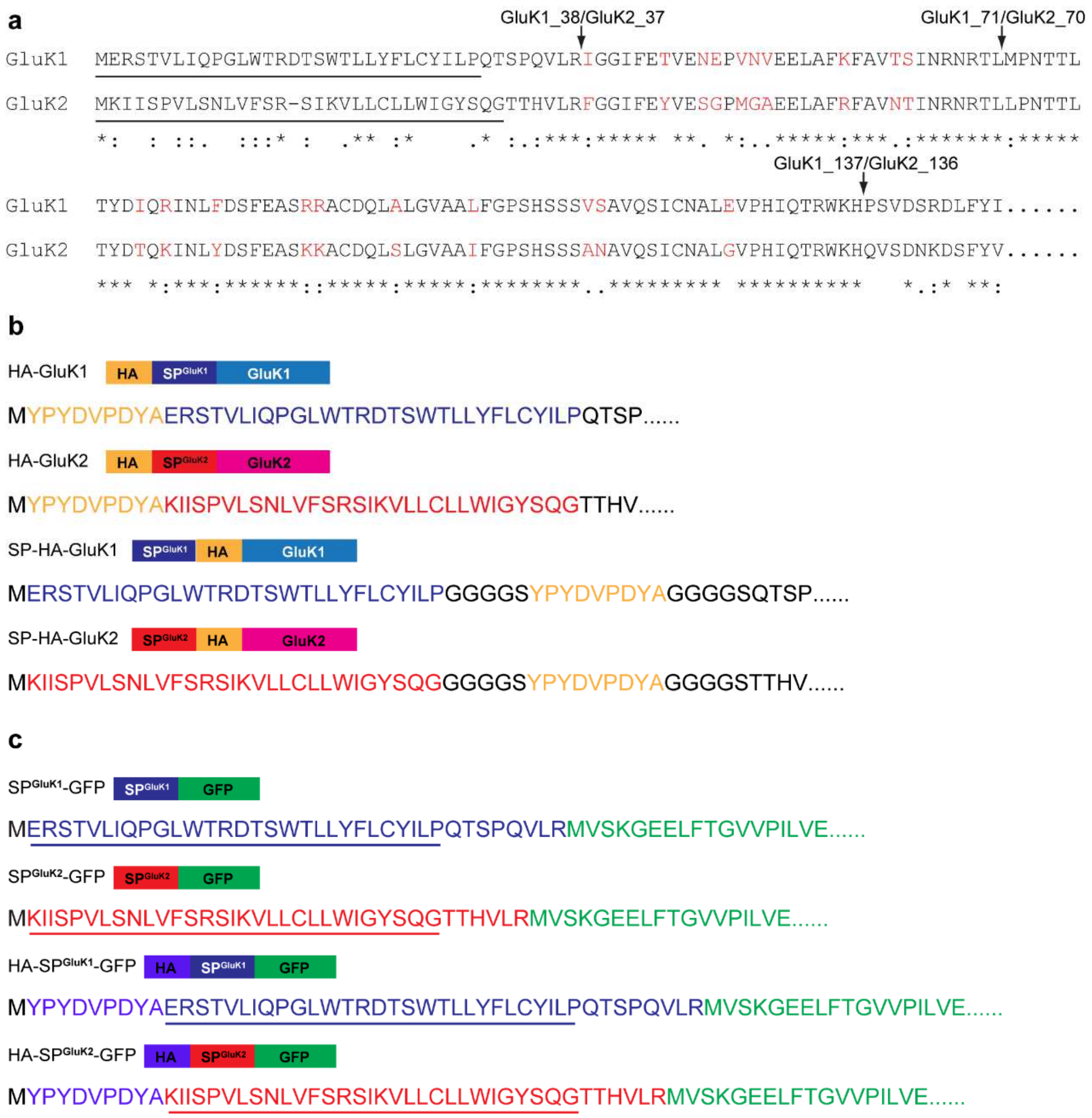

Supplementary Figure 1. Amino acid sequences of key molecules used in the study. **a** The aligned protein sequences around the signal peptide region of GluK1 and GluK2 receptors. The highlighted amino acids in red are different residues between the two receptors and are used for the mutants in the main text. Underlined are signal sequences. **b, c** The sequences of the HA tag and the signal peptides of GluK1 or GluK2 in the indicated tethered proteins used in Fig. 2.

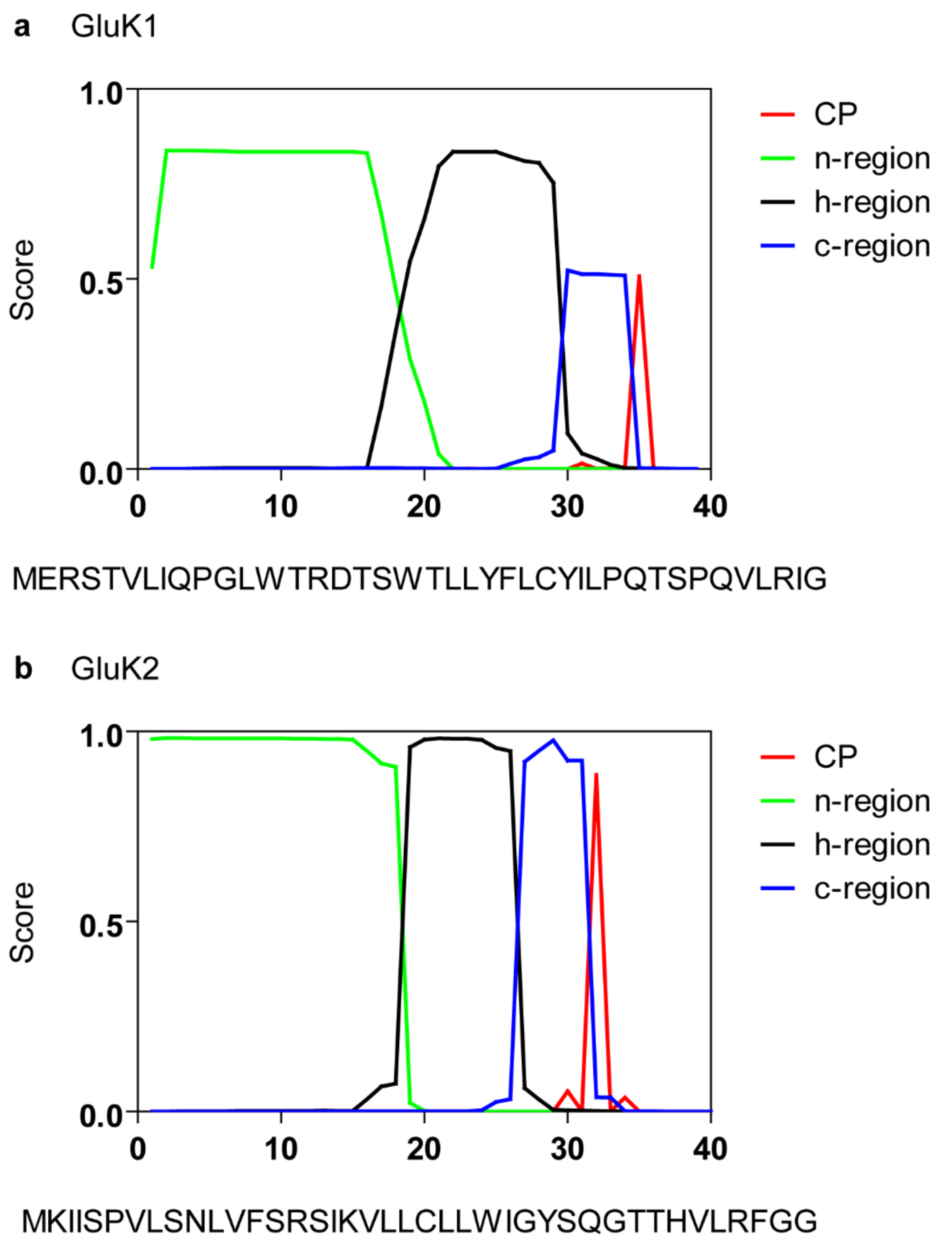

Supplementary Figure 2. The cleavage probability of GluK1 and GluK2 signal peptides. a, b Signal peptide cleavage prediction results of GluK1 and GluK2. Cleavable signal peptide of eukaryotic membrane proteins shares similar features: a hydrophobic α-helical core (h region) flanked by rather polar amino acid residues (n region), and a C-terminal (c region). The tripartite structure of signal peptide is shown in three colors: a hydrophobic α-helical core (h region, green), flanked by rather polar amino acid residues (n region, black), and a C-terminal (c region, blue). The cleavage probabilities (cp, red) are indicated in a score ranging from 0 to 1. Predictions were carried out using the online program SignalP 3.0.

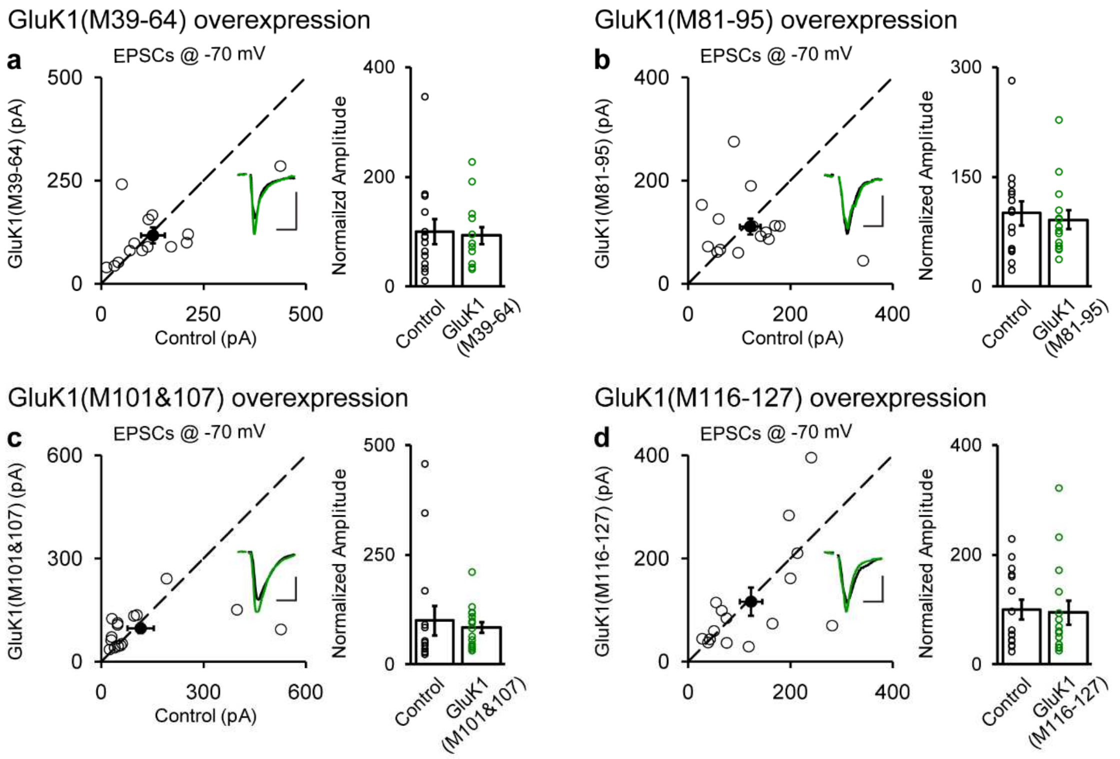

Supplementary Figure 3. Synaptic targeting ability of Mutated GluK1 receptors. Synaptic responses (eEPSCs measured at -70 mV) of GluK1 mutations in CA1 pyramidal neurons. **a** GluK1(M39-64) (the AAs 39-64 in GluK1 mutated to corresponding sequence in GluK2), n=14, 92.65 ± 15.32% control, $p > 0.05$; **b** GluK1(M81-95), n=15, 91.14 ± 12.60% control, $p > 0.05$; **c** GluK1(M101&107), n=15, 83.72 ± 12.47% control, $p > 0.05$; **d** GluK1(M116-127), n=15, 94.39 ± 22.15% control, $p > 0.05$). All the statistical analyses are comparisons of transfected neurons to respective control neurons with two-tailed Wilcoxon signed-rank sum test.

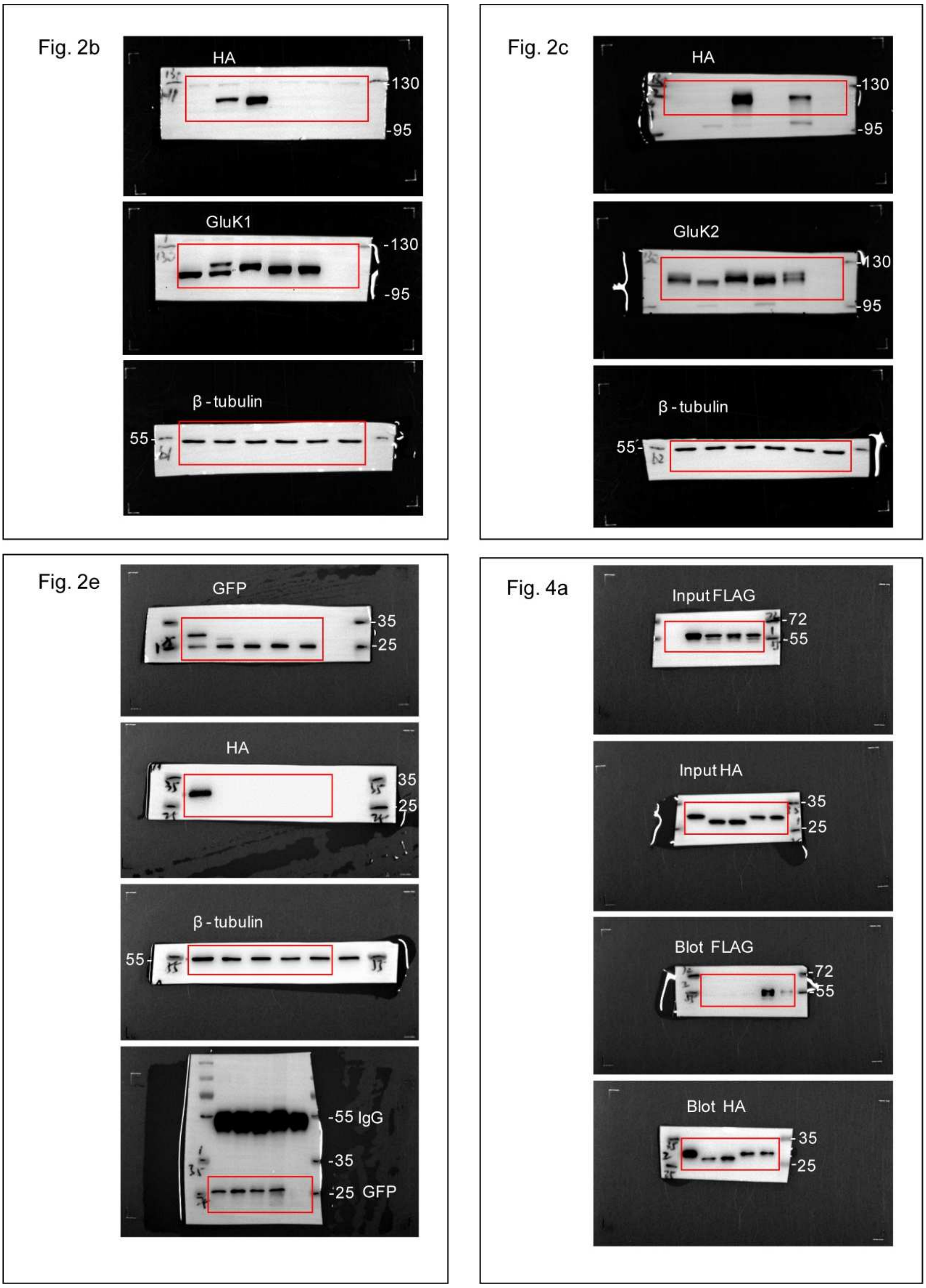

Supplementary Figure 4. The uncropped scans of western blots including the molecular weight markers for Figure 2b, Figure 2c, Figure 2e and Figure 4a as indicated.

Supplementary Table 1: primers for expression constructs in this study

pCAGGS-GluK1/GluK2-IRES-GFP primers	
GluK1-FW	GGACTCAGATCTCGAGATGGAGCGCAGCACAGTCCTT
GluK1-RV	GAAGCTTGAGCTCGAGTCACGCCACTGTCTCTTTTCT
GluK2-FW	GGACTCAGATCTCGAGATGAAGATTATTTCCCCAGTT
GluK2-RV	GAAGCTTGAGCTCGAGTCATGCCATGGTTTCTTTACC
pCAGGS-GFP-IRES-GluK1/GluK2 primers	
GluK1-FW	AATATGGGCCCAGAACCTTGGATGGAGCGCAGCACAGTC
GluK1-RV	GTATTTGTGAGCCAGGATCTTGGTCACGCCACTGTCTCTTT
GluK2-FW	ATAATATGGGCCCAGAACCTTGGATGAAGATTATTTCC
GluK2-RV	GTATTTGTGAGCCAGGATCTTGGTCATGCCATGGTTTC
GluK1&GluK2 chimera mutants primers	
GluK1(ATR^{GluK2}) FW	TTGATTGTTACCACCATTCTGGAAGAGCCC
GluK1(ATR^{GluK2}) RV	GGGCTCTTCCAGAATGGTGGTAACAATCAA
GluK2(ATR^{GluK1}) FW	CTCATTGTCACCACTATTTTGGAAGAACCG
GluK2(ATR^{GluK1}) RV	CGGTTCTTCCAAAATAGTGGTGACAATGAG
GluK1(SP^{GluK2}) FW	CAAGGAACCACACATGTGCTCAGGATCGGA
GluK1(SP^{GluK2}) RV	TCCGATCCTGAGCACATGTGTGGTTCCTTG
GluK2(SP^{GluK1}) FW	CAGACCTCCCCTCAAGTGTTAAGATTCGGT
GluK2(SP^{GluK1}) RV	ACCGAATCTTAACACTTGAGGGGAGGTCTG
GluK1(SP^{GluA1}) FW	GTGGGTGCCAATTTCCAAGTGCTCAGGATC
GluK1(SP^{GluA1}) RV	GATCCTGAGCACTTGGAAATTGGCACCCAC
GluK2(N71^{GluK1}) FW	ATTAACCGAAACCGAACTTTGCTGCCCAAC
GluK2(N71^{GluK1}) RV	GTTGGGCAGCAAAGTTCGGTTTCGGTTAAT
GluK2(N137^{GluK1}) FW	GTTCCACACATTCAGACCCGCTGGAAGCAC
GluK2(N137^{GluK1}) RV	GTGCTTCCAGCGGGTCTGAATGTGTGGAAC
GluK1(N136^{GluK2}) FW	GTTCCCCACATACAGACTCGCTGGAAACAC
GluK1(N136^{GluK2}) RV	GTGTTTCCAGCGAGTCTGTATGTGGGGAAC
GluK1 site-mutation primers	
GluK1I39F-FW	CCTCAAGTGCTCAGGTTCGGAGGGATTTTT
GluK1I39F-RV	AAAAATCCCTCCGAACCTGAGCACTTGAGG
GluK1M45-53-FW	GGGATTTTTGAATATGTGGAAAGTGGACCTAT GGGTGCTGAAGAATTAGC
GluK1M45-53-RV	GCTAATTCTTCAGCACCCATAGGTCCACTTTC CACATATTCAAAAATCCC
GluK1M59-64-FW	GCTTTCAGGTTTGCAGTCAACACTATTAAC
GluK1M59-64-RV	GTTAATAGTGTTGACTGCAAACCTGAAAGC AGATTAATTTTCTGGGTG
GluK1M81-95-FW	CACCCAGAAAATTAATCTTTATGATAGTTTTG AAGCCTCCAAAAAAGCAT

GluK1M81-95-RV	ATGCTTTTTTGGAGGCTTCAAAACTATCATAAAGATTAATTTTCTGGGTG
GluK1M101&107-FW	CTGTCTCTCGGGGTGGCCGCAATCTTC
GluK1M101&107-RV	GAAGATTGCGGCCACCCCGAGAGACAG
GluK1M116-127-FW	TCCGCCAATGCTGTACAGTCTATTTGCAATGCTCTGGGAGTT
GluK1M116-127-RV	AACTCCCAGAGCATTGCAAATAGACTGTACAGCATTGGCGGA
pCAGGS-SP-HA-GluK1/GluK2-IRES-GFP primers	
GluK1-HA-FW	CCATACGACGTCCCAGACTACGCTGGCGGTGGAGGTAGTCAGACCTCCCCTCAAGTGC
GluK1-HA-RV	CTGGGACGTCGTATGGGTAACTACCTCCACCGCCAGGGAGGATGTAGCACAGG
GluK2-HA-FW	GTCCCAGACTACGCTGGCGGTGGAGGTAGTACCACACATGTGTTAAGATTCG
GluK2-HA-RV	GGACGTCGTATGGGTAACTACCTCCACCGCCTCCTTGCGAATATCCGATCC
pCAGGS-HA-GluK1/GluK2-IRES-GFP primers	
HA-GluK1-FW	CCGCTAGCGCCACCATGTACCCATACGACGTCCCAGACTACGCTGAGCGCAGCACAGTCCTT
HA-GluK2-FW	CCGCTAGCGCCACCATGTACCCATACGACGTCCCAGACTACGCTAAGATTATTTCCCCAGTT
pCAGGS-RV	GTACCGTCGACTGCAGAATTC
pCAGGS-HA-$SP^{GluK1/GluK2}$-GFP primers	
SP^{GluK1}-GFP-FW	AGTGCTCAGGATGGTGAGCAAGGGCGAGG
SP^{GluK1}-GFP-RV	TGCTCACCATCCTGAGCACTTGAGGGGAG
SP^{GluK2}-GFP-FW	TGTGCTCAGGATGGTGAGCAAGGGCGAGGAG
SP^{GluK2}-GFP-RV	TGCTCACCATCCTGAGCACATGTGTGGTTCC
pCAGGS-SP^{GluK2}-$ATD^{GluK1/GluK2}$-FLAG-IRES-mCHERRY primers	
pCAGGS-FW	CAACGTGCTGGTTATTGTG
ATD^{GluK1}-FLAG-RV	TCGAAGCTTGAGCTCGAGCTACTTATCGTCGTCATCCTTGTAATCGTTGGACCTGTCTCTGTTGCC
ATD^{GluK2}-FLAG-RV	TCGAAGCTTGAGCTCGAGCTACTTATCGTCGTCATCCTTGTAATCTGCTGGCTTTCCTTTCTGAC
pCAGGS-SP^{GluK2}-HA-GFP primers	
HA-GFP-FW	TACCCATACGACGTCCCAGACTACGCTGGCGGTATGGTGAGCAAGGGCGAG
HA-GFP-RV	TCTGGGACGTCGTATGGGTATCTTAACACATGTGTGGTTC
pCold-GST-$ATD^{GluK1/GluK2}$ primers	
ATD^{GluK1}-FW	CCGAATTCATGATCGGAGGGATTTTTGAAACTG
ATD^{GluK1}-RV	AGGTCGACCTAGTTGGACCTGTCTCTGTTGC
ATD^{GluK2}-FW	CCGAATTCATGACCACACATGTGTTAAGATTCG

ATD^{GluK2}-RV	AGGTCGACCTATGCTGGCTTTCCTTTCTGAC
pCold-GST-N72-137^{GluK1} primers	
N72-137^{GluK1}-FW	CCGAATTCATGATGCCCAATACCACATTAAC
N72-137^{GluK1}-RV	AGGTCGACCTAGTGTTTCCAGCGAGTCTGAATG
pCold-GST-N71-136^{GluK2}	
N71-136^{GluK2}-FW	CCGAATTCATGCTGCCCAACACCACTTTAAC
N71-136^{GluK2}-RV	AGGTCGACCTAGTGCTTCCAGCGGGTCTGTATG
pCold-GST-ATD^{GluA2}	
ATD^{GluA2}-FW	CCGAATTCATGCAAAAGATTATGCATATTTC
ATD^{GluA2}-RV	AGGTCGACCTAAGTCTTGTTTTCAAGCCC
